# Lack of pharmacokinetic drug–drug interaction between ramucirumab and paclitaxel in a phase II study of patients with advanced malignant solid tumors

**DOI:** 10.1007/s00280-016-3098-3

**Published:** 2016-07-05

**Authors:** Laura Q. M. Chow, David C. Smith, Antoinette R. Tan, Crystal S. Denlinger, Ding Wang, Dale R. Shepard, Archana Chaudhary, Yong Lin, Ling Gao

**Affiliations:** 1 University of Washington, Seattle Cancer Care Alliance, MSG4-940, 825 Eastlake Ave E, Box 358081, Seattle, WA 98109 USA; 2 University of Michigan, Ann Arbor, MI USA; 3 Rutgers Cancer Institute of New Jersey, New Brunswick, NJ USA; 4 Fox Chase Cancer Center, Philadelphia, PA USA; 5 Henry Ford Hospital, Detroit, MI USA; 6 Cleveland Clinic Foundation, Cleveland, OH USA; 7 Eli Lilly and Company, Indianapolis, IN USA; 8 Eli Lilly and Company, Bridgewater, NJ USA

**Keywords:** Ramucirumab, Paclitaxel, Pharmacokinetics, Drug–drug interactions, Cancer

## Abstract

**Purpose:**

The objective of this phase II study was to evaluate pharmacokinetic interaction potential between ramucirumab and paclitaxel in patients with advanced cancer.

**Methods:**

This study was designed to assess 2-way pharmacokinetic drug–drug interactions between ramucirumab and paclitaxel. Twenty-four patients participated in Part A, which consisted of a 2-week monotherapy period in which paclitaxel 80 mg/m^2^ was administered on day 1, followed by a 4-week cycle of combination treatment with ramucirumab (8 mg/kg on days 1 and 15; paclitaxel on days 1, 8, and 15). Patients could continue to receive combination therapy with ramucirumab and paclitaxel. In 16 patients in Part B, ramucirumab monotherapy was administered on day 1 of a 3-week cycle. Patients could continue to receive ramucirumab monotherapy or combination therapy with paclitaxel.

**Results:**

Concomitant administration of ramucirumab had no effect on pharmacokinetics of paclitaxel, with ratios of geometric least squares (LS) means (with ramucirumab vs. alone) of 1.09 (90 % confidence interval [CI] 0.93, 1.29) for AUC_(0–∞)_ and 0.97 (90 % CI 0.83, 1.13) for *C*_max_. In addition, similar ramucirumab pharmacokinetic characteristics were observed with or without paclitaxel administration. The ratios of geometric LS means of AUC_(0–∞)_ and *C*_max_ of ramucirumab (with paclitaxel vs. alone) were 1.00 (90 % CI 0.84, 1.19) for AUC_(0–∞)_ and 1.07 (90 % CI 0.93, 1.24) for *C*_max_, respectively.

**Conclusions:**

Concomitant paclitaxel administration is unlikely to affect the pharmacokinetics of ramucirumab, and vice versa. The incidence and severity of adverse events were consistent with the known safety profiles of paclitaxel and ramucirumab.

## Introduction

Pathways that mediate angiogenesis are considered important targets in cancer drug development. Vascular endothelial growth factors (VEGFs) have emerged as key regulators of angiogenesis. VEGF receptor 2 (VEGFR-2) is the primary mediator of proangiogenic effects of VEGF-A, and experimental evidence suggests that the VEGF-A/VEGFR-2 interaction plays an important role in tumor angiogenesis, a process essential for tumor growth and metastasis [1, 2]. Disruption of the interaction between VEGF-A and VEGFR-2 has proven to have therapeutic application in the treatment of cancer.

Ramucirumab is a recombinant human immunoglobulin G1 monoclonal antibody that specifically binds to the VEGFR-2 receptor with high affinity, preventing binding of VEGF-A, VEGF-C, and VEGF-D and inhibiting receptor activation [3, 4]. In patients with previously treated advanced gastric or gastroesophageal junction adenocarcinoma, the results from 2 randomized, phase III trials demonstrated that overall survival was significantly increased in patients who received ramucirumab as monotherapy (hazard ratio [HR] 0.78; 95 % confidence interval [CI] 0.60, 0.998; *P* = 0.047) and in combination with paclitaxel (HR 0.81; 95 % CI 0.68, 0.96; *P* = 0.017) [5, 6]. These trials led to approval of ramucirumab as second-line therapy for advanced gastric cancer.

Paclitaxel is a cytotoxic agent active against various types of malignancies and is used as a single agent or in combination with other agents for advanced gastric cancer [7, 8]. Paclitaxel is a microtubule inhibitor and is metabolized by cytochromes P450 (CYP) CYP3A4 and CYP2C8 [9, 10].

An increase in chemotherapy-associated toxicities such as neutropenia has been observed with the addition of anti-VEGF antibodies [11]. In ECOG 4599, a randomized phase III trial in non-squamous non-small cell lung cancer (NSCLC), patients received paclitaxel/carboplatin with or without bevacizumab [12]. In this trial, the rates (grade ≥4 hematologic; grade ≥3 non-hematologic) of hypertension, proteinuria, bleeding, neutropenia, febrile neutropenia, and thrombocytopenia were significantly higher in the paclitaxel/carboplatin/bevacizumab group than in the paclitaxel/carboplatin group (*P* < 0.05). The underlying mechanism of these events is unknown. An argument could be made whether the pharmacokinetics of chemotherapy can be impacted by coadministration with anti-angiogenic compounds.

To support the concomitant use of ramucirumab with paclitaxel for the treatment of advanced gastric cancer, this phase II study assessed the potential for pharmacokinetic drug–drug interactions (DDIs) between ramucirumab and paclitaxel when given in combination in patients with advanced malignant solid tumors.

## Methods

### Patients

This study was a 2-part, multicenter, open-label phase II study in patients with advanced solid tumors (ClinicalTrials.gov: NCT01515306). Eligible patients were 18 years of age or older; had metastatic or locally advanced malignant solid tumors that were resistant to standard therapy or for which no standard therapy was available; had adequate organ and hematologic function; had no history of uncontrolled hypertension or bleeding; and had an ECOG PS of 0–2. For Part A only, patients were required to have had 1 or fewer prior taxane-containing treatment regimens (including taxane monotherapy), which should have been completed at least 6 months before the first dose of study drug. Prior treatment with bevacizumab was allowed. For Part B only, prior bevacizumab and taxane-containing treatment regimens (including taxane monotherapy) were allowed and should have been completed at least 6 months before the first dose of study drug. The study was undertaken in accordance with principles of the Declaration of Helsinki and Good Clinical Practice guidelines and with local institutional review board approval. Written informed consent was obtained from all participants.

### Treatment

This study was designed to assess 2-way pharmacokinetic DDIs between ramucirumab and paclitaxel.

Part A consisted of a 2-week monotherapy period (Cycle 1), during which paclitaxel 80 mg/m^2^ was administered on day 1, followed by a 4-week cycle of combination treatment (Cycle 2). Ramucirumab (8 mg/kg) was administered on days 1 and 15, and paclitaxel (80 mg/m^2^) was administered on days 1, 8, and 15 of each 4-week treatment cycle. This was followed by continuation of the treatment phase (Cycle 3 and beyond), in which patients could continue to receive combination therapy with ramucirumab and paclitaxel.

In Part B, ramucirumab 8 mg/kg monotherapy was administered on day 1 of a 3-week cycle. This was followed by continuation of the treatment phase (Cycle 2 and beyond), in which patients could continue to receive ramucirumab 8 mg/kg monotherapy or combination therapy with paclitaxel.

All patients who completed Cycle 1, Day 1 and Cycle 2, Day 1 in Part A and Cycle 1, Day 1 in Part B were included in the DDI analysis.

### Pharmacokinetics

In Part A, on Day 1 of Cycle 1, blood samples for paclitaxel concentration analysis were drawn at time point 0 (immediately before initiation of paclitaxel infusion) and at 1, 1.5, 2, 5, 7, 24, 48, 72, and 168 h after the start of the paclitaxel infusion. For Cycle 2, blood samples for paclitaxel and ramucirumab concentration analysis were drawn at day 1, time point 0 (immediately before initiation of ramucirumab infusion) and at 1, 1.5, 2, 5, 7, 24, 48, 72, 96, 168, 264, and 336 h after the start of the ramucirumab infusion.

In Part B, on Day 1 of Cycle 1, blood samples for ramucirumab concentration analysis were drawn at time point 0 (immediately before initiation of ramucirumab infusion) and at 1, 1.5, 2, 5, 7, 24, 48, 72, 168, 264, 336, 408, and 504 h after the start of the ramucirumab infusion.

Plasma samples were analyzed for paclitaxel using a validated liquid chromatography tandem mass spectrometry method (PPD method LCMSC 163.4 version 1.00) at PPD (Richmond, VA, USA). Serum samples were analyzed for ramucirumab using a modified validated enzyme-linked immunosorbent assay (ELISA) method at Intertek Pharmaceutical Services (San Diego, California, USA).

Pharmacokinetic parameters were assessed for ramucirumab and paclitaxel and were calculated by standard non-compartmental methods of analysis using Phoenix^®^ WinNonlin^®^ Professional 6.2. Area under the curve versus time curve from *t* = 0 extrapolated to infinity (AUC_(0–∞)_) and *C*_max_ was dose normalized for the DDI comparisons because patients received different absolute doses.

### Statistical analyses

Log-transformed pharmacokinetic parameters of AUC_(0–∞)_ and *C*_max_ for paclitaxel were analyzed via a linear mixed-effects model containing treatment (paclitaxel; ramucirumab plus paclitaxel) as a fixed effect and patient as a random effect in Part A.

LS means and 90 % CIs for the differences between AUC_(0–∞)_ and *C*_max_ of paclitaxel in log scale between Cycle 1 and 2 were estimated before transformation back to the original scale to estimate the ratio of geometric means and 90 % CIs for the comparisons (ramucirumab plus paclitaxel vs. paclitaxel).

A 2-sample *t* test was used to analyze the log-transformed pharmacokinetic parameters of AUC_(0–∞)_ and *C*_max_ for ramucirumab when coadministered with paclitaxel (Part A) and for ramucirumab monotherapy (Part B).

All calculations were performed using SAS^®^ version 9.2.

### Safety

All patients receiving at least one dose of a study drug were included in the summary and analysis of safety in Parts A and B of the study. All enrolled patients were assessed for toxicity before each infusion using National Cancer Institute Common Terminology Criteria for Adverse Events (NCI-CTCAE) v. 4.0.

## Results

### Patient demographics and disease characteristics

#### Part A

Twenty-four patients (11 males [45.8 %] and 13 females [54.2 %]) between the ages of 23 and 83 years (median age 60.5 years) participated in Part A of this study (Table 1). The majority of patients had an Eastern Cooperative Oncology Group (ECOG) performance status (PS) of 0 or 1 (20 patients, 83.3 %). Four patients (16.7 %) had an ECOG PS of 2. Breast carcinoma (4 patients, 16.7 %) followed by NSCLC, soft tissue sarcoma, and urothelial carcinoma (3 patients each, 12.5 %) were the most commonly reported sites of origin for the primary tumor.

**Table 1:** Patient demographics and disease characteristics

Characteristic	Part A (*n* = 24)	Part B (*n* = 16)
Gender, *n* (%)		
Male	11 (46)	7 (44)
Female	13 (54)	9 (56)
Age, years		
Median, (range)	60.5 (23–83)	61.0 (19–83)
<65, *n* (%)	14 (58)	10 (63)
≥65, *n* (%)	10 (42)	6 (38)
Race, *n* (%)		
Asian	1 (4)	1 (6)
White	22 (92)	15 (94)
Multiple	1 (4)	0
Ethnicity, *n* (%)		
Hispanic or Latino	1 (4)	0
Non-Hispanic or Latino	23 (96)	16 (100)
ECOG PS, *n* (%)		
0	6 (25)	9 (56)
1	14 (58)	6 (38)
2	4 (17)	1 (6)
Duration of disease, months		
Median (range)	35 (3–174)	17 (2–64)
Prior (21 days) anticancer treatment, *n* (%)	1 (4)	0
Prior (14 days) radiotherapy, *n* (%)	3 (13)	0
Prior taxane therapy, *n* (%)	2 (8)	0
Type of cancer,^a^ *n* (%)		
Breast	4 (17)	0
Non-small cell lung	3 (13)	2 (13)
Sarcoma, soft tissue	3 (13)	4 (25)
Urothelial	3 (13)	0
Hepatobiliary	0	2 (13)

*ECOG PS* Eastern Cooperative Oncology Group performance status

^a^Part A, one patient each (4.2 %): colorectal, gastric, melanoma, mesothelioma, neuroendocrine tumor, ovarian, cholangiocarcinoma, chondrosarcoma, leiomyosarcoma, squamous cell carcinoma of the tonsil, unknown primary site. Part B, one patient each (6.3 %): mesothelioma, neuroendocrine tumor, pancreatic, small cell lung, urothelial, extrapulmonary small cell, large cell neuroendocrine tumor of the lung, mucinous biliary cyst adenocarcinoma

#### Part B

Part B comprised 16 patients (7 males [43.8 %] and 9 females [56.3 %]) between the ages of 19 and 83 years (median age 61.0 years). The majority of patients in Part B had an ECOG PS of 0 or 1 (15 patients, 93.8 %). Only one patient (6.3 %) had an ECOG PS of 2. Soft tissue sarcoma (4 patients, 25.0 %) followed by hepatobiliary carcinoma and NSCLC (2 patients each, 12.5 %) were the most commonly reported sites of origin for the primary tumor.

### Pharmacokinetic analysis

Concentrations of paclitaxel and ramucirumab were determined in 23 patients in Part A and 16 patients in Part B. No pharmacokinetic parameters were calculated for one patient in Part A because of an infusion duration of approximately 4 h.

#### Paclitaxel pharmacokinetics

**Fig. 1:**
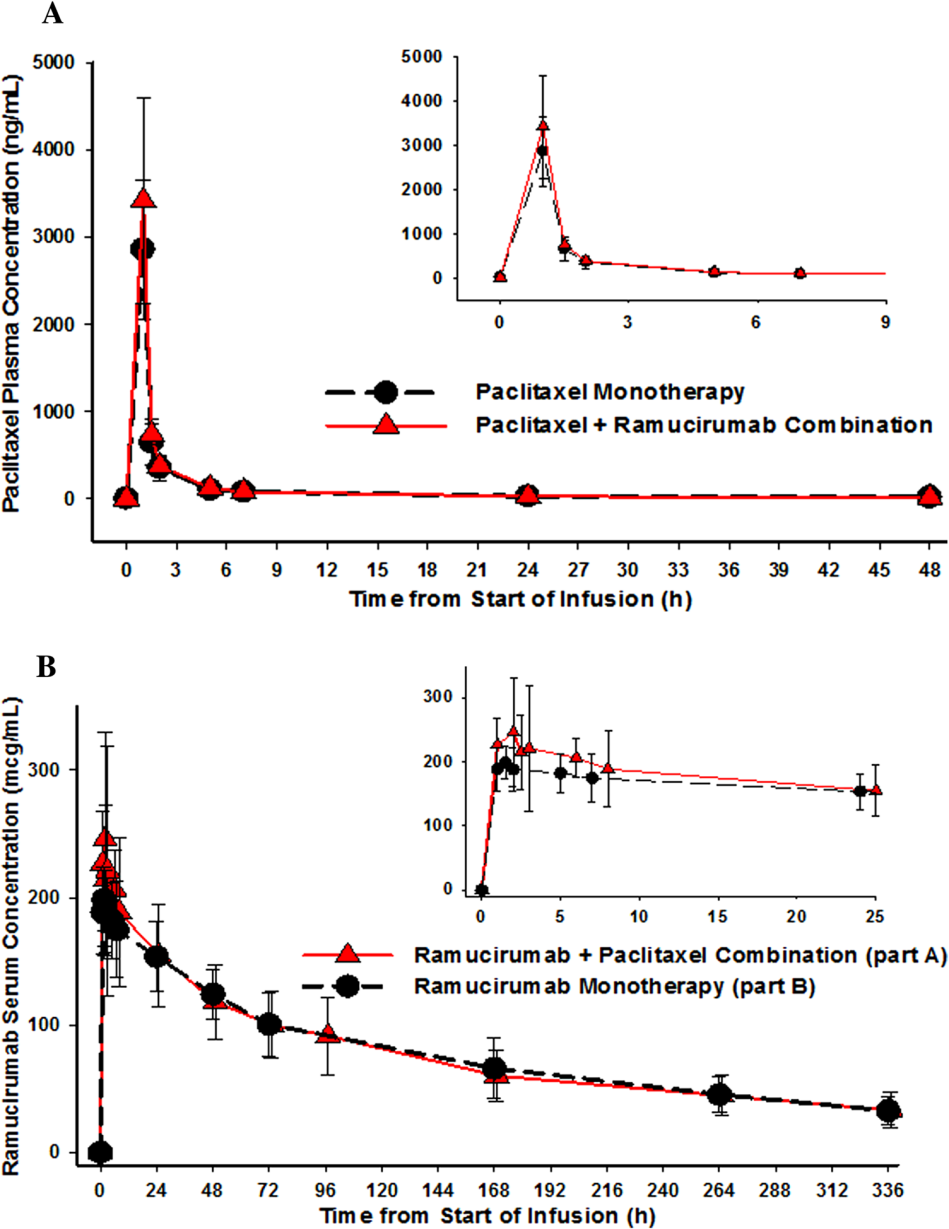
Mean (±SD) plasma concentration–time profile of paclitaxel from Part A, Cycle 1 (monotherapy) and Cycle 2 (combination therapy); inset: concentration curve from 0 to 9 h (**a**). Mean (±SD) serum concentration–time profile of ramucirumab from Part A, cycle 2 (combination therapy), and Part B, cycle 1 (monotherapy); inset: concentration curve from 0 to 25 h (**b**). *H* hour, *SD* standard deviation

Figure 1a shows mean plasma concentrations of paclitaxel over time after monotherapy (Part A, Cycle 1) and combination therapy (Part A, Cycle 2). Overlapped paclitaxel pharmacokinetic profiles were observed between monotherapy and combination therapy. Maximum paclitaxel plasma concentrations were achieved at the end of paclitaxel infusion and then declined in a biexponential fashion, consistent with previously reported profiles [13, 14]. Statistical analysis demonstrated that coadministration of ramucirumab had no effect on dose-normalized area under the curve (AUC_(0–∞)_) and maximum drug concentration (*C*_max_) of paclitaxel; ratios (Part A, Cycle 2 vs. Cycle 1) of geometric least squares (LS) means were 1.09 (90 % CI 0.93, 1.29) and 0.97 (90 % CI 0.83, 1.13), respectively (Table 2). Other pharmacokinetic parameters were also similar between paclitaxel monotherapy and in combination with ramucirumab (Table 3).

**Table 2:** Drug–drug interaction assessment

Analyses and PK parameters^a^	*n*	Geometric LSM (90 % CI)	*n*	Geometric LSM (90 % CI)	Ratio of geometric LSM (90 % CI)
The effect of coadministration of ramucirumab on paclitaxel		Paclitaxel alone cycle 1 Part A		Ramucirumab+ paclitaxel cycle 2 Part A	Ramucirumab+ paclitaxel: paclitaxel
AUC_(0-∞)_ (ng h/mL/mg)	19	29.00 (24.50, 34.34)	17	31.67 (26.58, 37.73)	1.09 (0.93, 1.29)
*C*_max_ (ng/mL/mg)	21	18.84 (16.03, 22.13)	20	18.30 (15.54, 21.56)	0.97 (0.83, 1.13)
The effect of coadministration of paclitaxel on ramucirumab		Ramucirumab alone cycle 1 Part B		Ramucirumab+ paclitaxel cycle 2 Part A	Ramucirumab+ paclitaxel: ramucirumab
AUC_(0–∞)_ (ng h/mL/mg)	15	55.32 (48.97, 62.50)	13	55.43 (48.63, 63.18)	1.00 (0.84, 1.19)
*C*_max_ (ng/mL/mg)	16	0.36 (0.33, 0.39)	21	0.38 (0.34, 0.43)	1.07 (0.93, 1.24)

*AUC*_(*0*–*∞*)_ area under the plasma concentration versus time curve from time zero extrapolated to infinity, *CI* confidence interval, *C*_*max*_ maximum plasma drug concentration, *LSM* least squares mean, *n* number of patients included in the analysis, *PK* pharmacokinetics

^a^For the population of patients who completed Cycle 1, Day 1 and Cycle 2, Day 1

**Table 3:** Paclitaxel pharmacokinetic parameters as monotherapy or as combination therapy

Parameter	Geometric mean (CV%)	
	Paclitaxel alone (cycle 1) (*n* = 23)	Paclitaxel + ramucirumab (cycle 2) (*n* = 20^a^)
*C*_max_ (ng/mL)	2742.61 (30)	2662.40 (47)
Dose-normalized *C*_max_ (ng/mL/mg)	19.1 (33)	18.5 (54)
*t*_1/2_^b^ (h)	11.4^c^ (8.26–18.9)	11.4^d^ (6.97–15.6)
AUC_(0–∞)_ (ng h/mL)	4280^c^ (29)	4560^d^ (46)
Dose-normalized AUC_(0–∞)_ (ng h/mL/mg)	29.7^c^ (36)	31.4^d^ (52)
CL (L/h)	33.7^c^ (36)	31.9^d^ (52)
*V*_ss_ (L)	241^c^ (35)	226^d^ (72)

*AUC*_*(0*–*∞)*_ area under the plasma concentration versus time curve from time zero extrapolated to infinity, *C*_*max*_ maximum plasma drug concentration, *CL* clearance, *CV%* percentage coefficient of variation, *n* number of subjects who had data for calculation of at least one pharmacokinetic parameter, *t*_*1/2*_ terminal half-life, *V*_*ss*_ volume of distribution at steady state following intravenous administration

^a^21 subjects completed Cycle 2, Day 1, but no pharmacokinetic parameters were calculated for one patient because of an infusion duration of 4.32 h

^b^Geometric mean (range)

^c^ *n* = 21

^d^ *n* = 17

#### Ramucirumab pharmacokinetics

As shown in Fig. 1b, mean serum concentrations of ramucirumab over time in combination with paclitaxel (Part A, Cycle 2) and as monotherapy (Part B, Cycle 1) were similar. Ramucirumab pharmacokinetic parameters between monotherapy and in combination with paclitaxel were also comparable (Table 4). Statistical analysis was performed to assess the effect of coadministration of paclitaxel on the pharmacokinetics of ramucirumab. Dose-normalized AUC_(0–∞)_ and *C*_max_ of ramucirumab in Cycle 2 of Part A were similar to those when ramucirumab was administered alone in Cycle 1 of Part B, with ratios (Part A, Cycle 2 vs. Part B, Cycle 1) of geometric means of 1.00 (90 % CI 0.84, 1.19) for AUC_(0–∞)_ and 1.07 (90 % CI 0.93, 1.24) for *C*_max_ (Table 2).

**Table 4:** Ramucirumab pharmacokinetic parameters as combination therapy or as monotherapy

Parameter	Geometric mean (CV%)	
	Part A	Part B
	Paclitaxel + ramucirumab (cycle 2) (*n* = 21)	Ramucirumab alone (cycle 1) (*n* = 16)
*C*_max_ (μg/mL)	216.41 (24)	205.71 (14)
Dose-normalized *C*_max_ (μg/mL/mg)	0.384 (31)	0.358 (18)
*t*_1/2_^a^ (h)	139^b^ (78.5–193)	157^c^ (77.9–241)
AUC_(0–∞)_ (μg h/mL)	29100^b^ (28)	32100^c^ (29)
Dose-normalized AUC_(0–∞)_ (μg h/mL/mg)	55.4^b^ (27)	55.3^c^ (27)
CL (L/h)	0.018^b^ (27)	0.018^c^ (27)
*V*_ss_ (L)	3.41^b^ (23)	3.95^c^ (23)

*AUC*_*(0*–*∞)*_ area under the plasma concentration versus time curve from time zero extrapolated to infinity, *C*_*max*_ maximum plasma drug concentration, *CL* clearance, *CV* *%* percentage coefficient of variation, *n* number of subjects who had data for calculation of at least one pharmacokinetic parameter, *t*_*1/2*_ terminal half-life, *V*_*ss*_ volume of distribution at steady state following intravenous administration

^a^Geometric mean (range)

^b^ *n* = 13

^c^ *n* = 15

### Safety

#### Part A

In Part A, paclitaxel was administered in Cycle 1 (*n* = 24), and ramucirumab plus paclitaxel was administered in Cycle 2 + (*n* = 21). In Cycle 1, 2 patients (8.3 %) discontinued due to a treatment-emergent adverse event (TEAE): 1 patient discontinued due to grade 3 increased hepatic enzymes, and 1 patient discontinued due to a grade 2 infusion-related reaction. Two patients (8.3 %) had a reduced or interrupted dose in Cycle 1 due to neutropenia and infusion-related reaction. In Cycle 2 or beyond, 1 (4.8 %) patient discontinued study treatment due to grade 3 anemia. In the safety population in Part A, 10 patients (41.7 %) had a TEAE leading to dose reduction or interruption, 7 (29.2 %) of which were grade 3. Reasons for dose modifications included grade 3 neutropenia, paresthesia, hypophosphatemia, influenza and grade 2 neutropenia and infusion-related reaction. TEAEs occurring in at least 10 % of patients are summarized in Table 5. Overall, the most frequent any grade TEAEs were fatigue (12 patients, 50.0 %), anemia (10 patients, 41.7 %), and diarrhea, decreased appetite, and epistaxis (7 patients each, 29.2 %).

**Table 5:** Treatment-emergent adverse events occurring in at least 10 % of patients

Preferred term	Part A (*n* = 24)		Part B (*n* = 16)	
	Any grade *n* (%)	Grade ≥3 *n* (%)	Any grade *n* (%)	Grade ≥3 *n* (%)
Fatigue	12 (50)	0	2 (13)	1 (6)
Anemia	10 (42)	1 (4)	0	0
Diarrhea	7 (29)	0	2 (13)	0
Decreased appetite	7 (29)	1 (4)	0	0
Epistaxis	7 (29)	0	4 (25)	0
Alopecia	6 (25)	0	0	0
Constipation	6 (25)	0	0	0
Nausea	6 (25)	0	3 (19)	0
Dizziness	5 (21)	0	0	0
Dyspnea	5 (21)	0	0	0
Headache	5 (21)	0	2 (13)	0
Hypertension	5 (21)	2 (8)	0	0
Pyrexia	5 (21)	0	0	0
Abdominal pain	4 (17)	0	2 (13)	0
Back pain	4 (17)	2 (8)	0	0
Cough	4 (17)	1 (4)	0	0
Dysgeusia	4 (17)	0	0	0
Myalgia	4 (17)	0	0	0
Neuropathy peripheral	4 (17)	0	0	0
Neutropenia	4 (17)	3 (13)	0	0
Edema peripheral	4 (17)	0	0	0
Upper respiratory tract infection	4 (17)	0	0	0
Vomiting	4 (17)	0	0	0
Arthralgia	3 (13)	0	0	0
Dry skin	3 (13)	0	0	0
Flushing	3 (13)	0	0	0
Infusion-related reaction	3 (13)	0	0	0
Pruritus	3 (13)	0	0	0
Stomatitis	3 (13)	0	2 (13)	0
Vision blurred	3 (13)	0	0	0
Weight decreased	3 (13)	0	0	0
Hyperglycemia	0	0	2 (13)	0
Insomnia	0	0	3 (19)	0
Decreased appetite	0	0	2 (13)	0
Dry mouth	0	0	2 (13)	0
Musculoskeletal pain	0	0	2 (13)	0
Stomatitis	0	0	2 (13)	0
Urinary tract infection	0	0	2 (13)	0

In Part A, adverse events of special interest (AESI) for patients who received ramucirumab in Cycle 2 and beyond included 8 patients (38.1 %) who experienced bleeding/hemorrhagic events, including epistaxis (7 patients, 33.3 %) and hemoptysis in 2 patients (9.5 %), 1 with colorectal cancer, and 1 with ovarian cancer. None of these events were grade 3 or greater. Five patients (23.8 %) experienced hypertension. Of these, 2 patients (9.5 %) experienced grade 3 hypertension; 1 of these 2 patients had a prior history of hypertension. One patient (4.8 %) experienced grade 3 pulmonary embolism.

Five of 24 patients (20.8 %) experienced serious adverse events (SAEs) during Part A. Of these, 3 patients (12.5 %) experienced SAEs that were considered related to study treatment. Three patients (12.5 %) died due to progressive disease, and 1 patient (4.2 %) died during Cycle 1 in Part A due to an AE that was not considered related to study treatment but to underlying disease progression.

#### Part B

Sixteen patients in Part B received ramucirumab monotherapy in Cycle 1; patients were allowed to continue to receive ramucirumab alone or in combination with paclitaxel. No patient discontinued from the study due to TEAEs in Cycle 1. One patient experienced an AE, leading to dose reduction or interruption in Cycle 2 + due to an infusion-related reaction. The most frequent any grade TEAEs were epistaxis (4 patients, 25.0 %) and nausea (3 patients, 18.8 %; Table 5). Six patients (37.5 %) experienced AESIs in Part B. Four patients (25.0 %) experienced bleeding/hemorrhagic events (epistaxis). One patient each (6.3 %) experienced mild proteinuria and increased blood creatinine levels. None of these events were grade 3 or greater. No SAEs or deaths occurred in Part B.

## Discussion

The primary objective of this phase II trial was to evaluate potential pharmacokinetic DDIs between ramucirumab and paclitaxel in patients with advanced malignant solid tumors resistant to standard therapy or for whom standard therapy was no longer available. The results demonstrated that no clinically meaningful changes in paclitaxel or ramucirumab exposure were observed when ramucirumab 8 mg/kg and paclitaxel 80 mg/m^2^ were coadministered in patients with solid tumors.

As paclitaxel is mainly metabolized by hepatic cytochrome P450 enzymes and monoclonal antibodies are eliminated through Fc receptor-mediated immunoglobulin clearance mechanisms and specific target-mediated drug disposition pathways, this finding was not unexpected [10, 15]. In DDI studies with other biologics, similar results were shown. A phase II study evaluating pharmacokinetics and safety found that the combination of paclitaxel and trastuzumab was generally well tolerated, with no unexpected toxicities and no pharmacokinetic interactions in women with human epidermal growth factor receptor 2-overexpressing metastatic breast cancer [16]. Likewise, no pharmacokinetic interactions were found with cetuximab in combination with paclitaxel and carboplatin, and the combination was safe and well tolerated in a population of stage IV NSCLC patients [17].

Systemic taxane-based chemotherapy is commonly used for patients with advanced disease. In a pivotal phase III study (RAINBOW), ramucirumab demonstrated significant improvement in overall survival in combination with paclitaxel versus paclitaxel alone in previously treated patients with advanced gastric cancers [6]. The current study was conducted to primarily support the use of ramucirumab in combination with paclitaxel for treatment in this patient population. Other studies have demonstrated that ramucirumab significantly improved overall survival as monotherapy; in combination with docetaxel; or in combination with irinotecan, folinic acid, and 5-fluorouracil (FOLFIRI) as second-line therapy in gastric, NSCLC, and metastatic colorectal cancer, respectively [5, 18, 19]. Two phase II studies evaluating the effect of concomitant ramucirumab on the pharmacokinetics of docetaxel or FOLFIRI in patients with advanced malignant solid tumors demonstrated no DDI between ramucirumab and these agents [20, 21].

The principal hematologic toxicity associated with paclitaxel is neutropenia [9]. In the RAINBOW trial, the incidence of grade 3 or 4 neutropenia was higher in the ramucirumab/paclitaxel group (grade 3, 71 patients [22 %]; grade 4, 62 patients [19 %]) versus the placebo/paclitaxel group (grade 3, 51 patients [16 %]; grade 4, 11 patients [3 %]) [6]. In this study, grade ≥3 neutropenia occurred in 3 patients (12.5 %) in Part A, and no patients in Part B. Results from this current study suggest that a synergistic effect of ramucirumab on paclitaxel-induced neutropenia is unlikely due to pharmacokinetic interactions.

No unexpected TEAEs or SAEs were observed with ramucirumab monotherapy or in combination with paclitaxel. The incidence and severity of TEAEs were consistent with the known safety profiles of paclitaxel and ramucirumab. Thus, these results demonstrate that no starting dose adjustments are needed for ramucirumab when coadministered with paclitaxel 80 mg/m^2^, and no starting dose adjustments are needed for paclitaxel when coadministered with ramucirumab 8 mg/kg, due to concerns for DDIs.
